# Research Note: Comparing methods to assess Valgus-Varus deformity in broiler chickens

**DOI:** 10.1016/j.psj.2022.101907

**Published:** 2022-04-06

**Authors:** H. van den Brand, R. Molenaar, M. Klaasen

**Affiliations:** ⁎ Adaptation Physiology Group, Wageningen University and Research, Wageningen, The Netherlands; † Veterinary Centre Someren, Someren, The Netherlands

**Keywords:** broiler, leg health, methodology, valgus-varus deformity

## Abstract

Valgus-varus deformity (**VVD**) is one of the leg disorders affecting health and welfare of broiler chickens. In research, several protocols are used to determine the prevalence and/or severity of VVD. This study aimed to investigate effects of five different protocols on the angulation of the tibiotarsal-tarsometatarsal joint. Angulation was determined (1) in living chickens with fixation at the femorotibiotarsal joint; (2) in dead chickens without fixation; (3) in dead chickens with fixation; (4) in dissected legs, including muscles, but without skin; (5) in dissected legs, without muscles, but with intact joints. Fixation of the leg at the femorotibiotarsal joint largely reduced the angulation of the tibiotarsal-tarsometatarsal joint. When fixation was used, no differences in angulation were found when broilers were live, dead or legs were dissected, but when no fixation was used, angulation was considerably higher, due to a large lateral deviation of the leg. It can be concluded that in intact chickens, fixation of the femorotibiotarsal joint is essential to determine VVD angulation in an appropriate way.

## INTRODUCTION

Valgus-varus deformity (**VVD**) is one of the leg disorders affecting health and welfare of broiler chickens, which can also lead to economic losses, due to culling, death or lower growth rate of affected chickens (Shim et al., 2012). VVD refers to an angular deviation of the tibiotarsal-tarsometatarsal joint, resulting in an outward (valgus) or inward (varus) deviation of the tarsometatarsus (Leterrier and Nys, 1992). The deformity is the result of a lateral or medial angulation of the shaft of the distal tibiotarsus, with similar, but less severe angulation in the proximal tarsometatarsus. In severe cases, it can be paralleled with flattened distal tibiotarsal condyles and/or displacement of the gastrocnemius tendon. VV angulation can occur bilaterally or unilaterally (Leterrier and Nys, 1992; Shim et al. 2012; Guo et al., 2019) and valgus occurs more often than varus in broiler chickens (Leterrier and Nys, 1992; Shim et al., 2012; González-Cerón et al., 2015; Martins et al., 2020). Incidence of VVD varies considerably among studies from 1.75% to 66.0% (Table 1). Possible reasons for this huge variation in VVD incidence among studies might be related to differences in growth rate, body weight, genetics, housing system, lighting schedule, age, litter quality, and diet composition (see Bradshaw et al., 2002 for review; Almeida Paz et al., 2010; González-Cerón et al., 2015; Martins et al., 2020). However, another contributing factor that might explain the variation in reported incidence rates is the method used to determine VVD. Leterrier and Nys (1992; Table 1, method 1) held living broilers at their wings and classified VVD in 4 categories, according to the angulation of the tibiotarsal-tarsometatarsal joint of the limb by visual appraisal. Angulation was classified as (1) normal (angle between tibiotarsus and tarsometatarsus <10^o^); (2) mild (angle between 10 and 25^o^); (3) intermediate (angle between 25 and 45^o^); and (4) severe (angle >45^o^). During VVD determination, it appears that legs were not fixated in this study. Almeida Paz et al. (2010; Table 1, method 2) determined the angle between the tibia and the third finger in living animals, using a calliper ruler and a protractor. In this study, it was not indicated how the animal was positioned or fixated during the measurement nor when an angle was classified as VVD. Güz et al. (2019; Table 1, method 3) determined VVD in dead broilers, using visual appraisal in nonfixated legs and classified all deviations from straight as VVD, whereas Van der Pol et al. (2017) did not describe their method at all. In the latter 2 studies also no method of positioning or fixating during measurements was provided. These differences in fixation and scoring of VVD makes it difficult or even impossible to compare VVD incidence among studies.

**Table 1:** Average incidence of varus and valgus deformities (%) in broiler chickens at slaughter age in different studies.

Study	Varus	Valgus	Method^1^
Leterrier and Nys (1992)	1–3	30–40	1
Shim et al. (2012)	3	29	1
Paz et al. (2013)	30*		2
González-Cerón et al. (2015)	4	26	1
Guo et al. (2019)	1.75*		1
Güz et al. (2019)	63–70*		3
Martins et al. (2020)	2	25	2

1 1 = Leterrier and Nys (1992), 2 = Almeida Paz et al. (2010), 3 = Güz et al., 2019.

⁎ Percentage of varus and valgus deformity taken together.

Aim of the current study was to compare different methods of measuring VV angulation in the same chickens. Our hypothesis was that depending on the method of fixation, the angulation of the tibiotarsal – tarsometatarsal joint varies and consequently the conclusion whether or not VVD is diagnosed might change.

## MATERIALS AND METHODS

In six male Ross 308 broiler chickens, reared under commercial conditions, at 38 d of age, VV angulation of the tibiotarsal-tarsometatarsal joint of both legs was determined with 5 different methods.

1. Living broiler, with the femorotibiotarsal joint fixated (Figure 1A).

**Figure 1:**
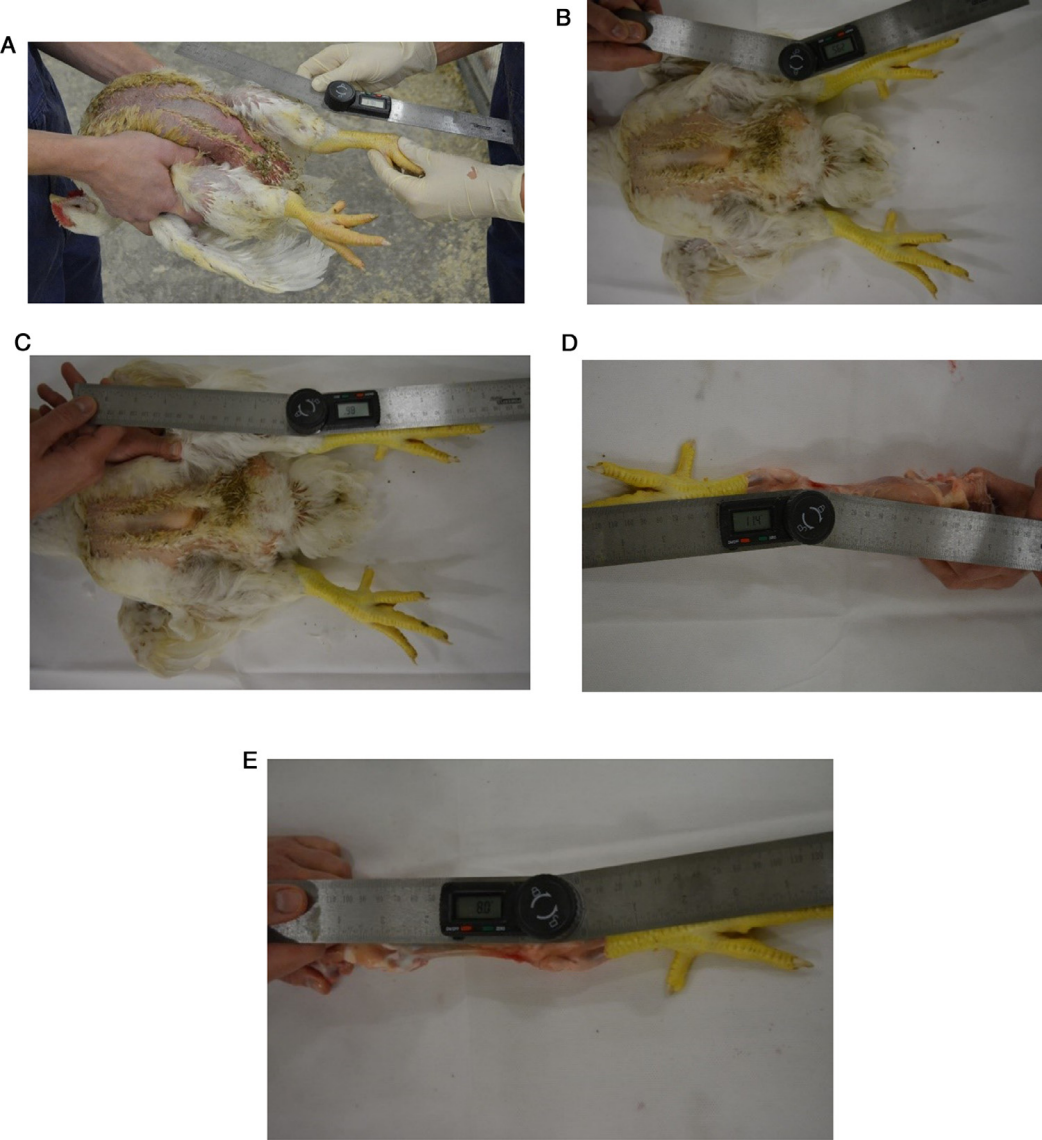
Determination of valgus-varus angulation with 5 different methods. A = Alive broiler, with femorotibiotarsal joint fixated; B = Dead broiler, without femorotibiotarsal joint joint fixated; C = Dead broiler, with femorotibiotarsal joint fixated; D = Dissected legs, skin removed, but intact muscles and joints, with the femorotibiotarsal joint fixated; E = Dissected legs, skin and musculature removed, but intact joints, with the femorotibiotarsal joint fixated.
2. Dead broiler, without the femorotibiotarsal joint fixated (Figure 1B).
3. Dead broiler, with the femorotibiotarsal joint fixated (Figure 1C).
4. Dissected legs, skin removed, but intact muscles and joints, with the femorotibiotarsal joint fixated (Figure 1D).
5. Dissected legs, skin and musculature removed, but intact joints, with the femorotibiotarsal joint fixated (Figure 1E).

**Figure 2:**
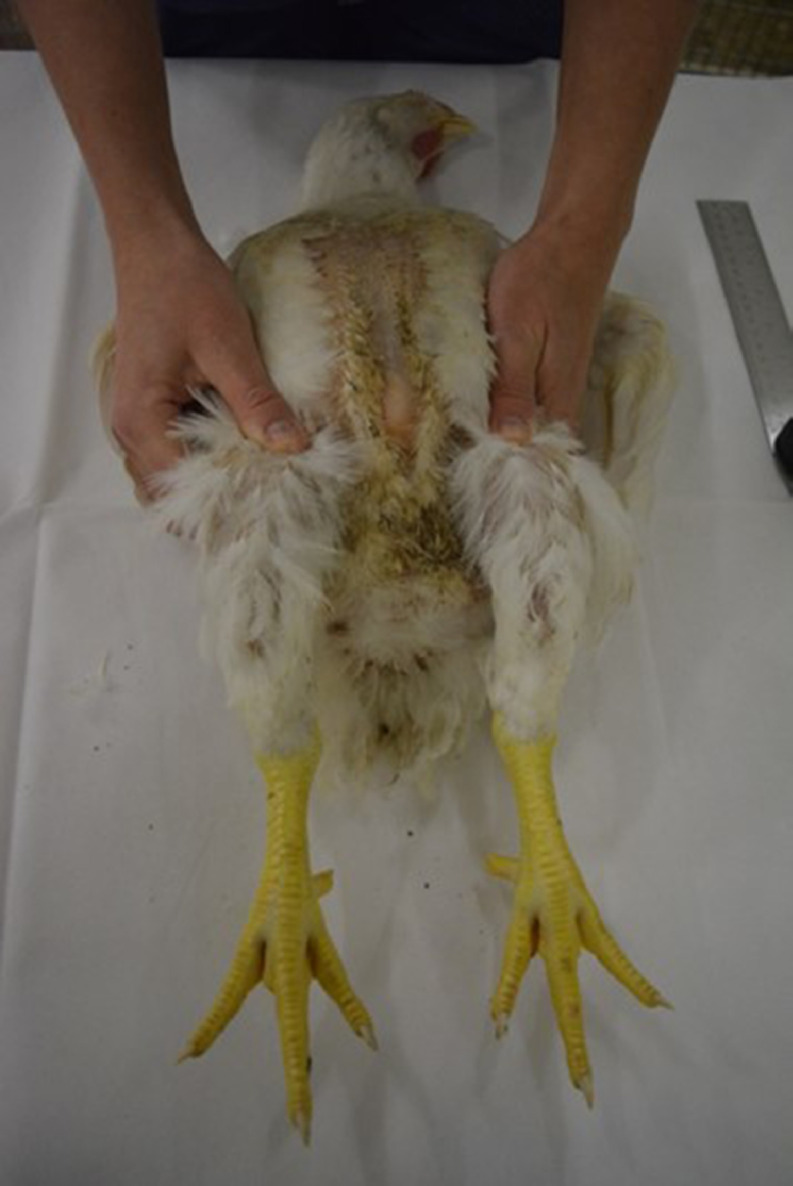
Fixation of the legs at the femorotibiotarsal (knee) joint.

The experimental protocol was approved by the Animal Use and Care committee of Wageningen University and Research. Chickens were obtained during regular postmortem control at the Veterinary Centre Someren (Someren, the Netherlands). Chickens were killed by a percussive blow on the head, according to EU directive 2010/63. In all 5 methods, angulation of the tibiotarsal-tarsometatarsal joint was determined three times per chicken per method, using a digital goniometer. One end of the goniometer was placed parallel along the tibiotarsus (from the middle of the femorotibiotarsal joint to the middle of the tibiotarsal-tarsometatarsal joint), whereas the other end of the goniometer was placed parallel along the middle of the tarsometatarsus (followed by the middle of the middle toe) (see Figure 1). All measurements were done by one single person and averaged per method used. In method 1, 3, 4 and 5, the legs were fixated by the same second person. This second person held and fixated both legs at the femorotibialtarsal (knee) joint, meaning that the legs were stretched as much as possible (see Figure 2).

VVD angulation was analysed with SAS (Version 9.4, 2013, SAS Institute Inc., Cary, North Carolina, US). Data was checked on normality of both means and residuals. Because VVD angulation was not normally distributed, a log transformation was performed before analysis.

VVD angulation was analysed with a MIXED procedure, using the model:

Y=μ+method+leg+interaction+e,

Where Y = VVD angulation, µ = overall mean, method = method of measuring (1–5), leg = leg (left, right), interaction = interaction between method and leg, e = residual error. Body weight was used as a covariate. Preliminary analysis did not show an interaction effect between method and leg and consequently this interaction was removed from the model. Chicken was used as the repeated subject and a compound symmetry covariance structure was applied.

Multiple comparisons were performed after correction for Bonferroni. Effects were considered to be significant at *P* ≤ 0.05.

## RESULTS

VV angulation was affected by method used, were the measurements on dead chickens without fixation of the femorotibiotarsal joint showed higher angulations than the other 4 methods (32.1 vs. 9.6^o^ on average; *P* < 0.001). No differences between left and right leg were observed (15.3 vs. 12.6° for left and right legs, respectively). In Table 2, data of the individual chickens per method is shown.

**Table 2:** Individual measurements of the angulation (in degrees) of the tibiotarsal-tarsometatarsal joint, using 5 different methods (means ± SE).

	Method^1^					
Chicken	Alive, fixed	Dead, non-fixed	Dead, fixed	Legs with muscles	Legs without muscles	BW, kg
1	5.0	22.0	6.0	2.0	4.0	2.55
2	6.5	29.0	8.0	9.0	9.0	3.10
3	7.5	36.5	8.5	11.5	8.0	2.56
4	13.0	35.5	12.5	13.0	9.5	3.03
5	21.5	45.5	17.5	18.0	14.0	2.41
6	6.0	19.0	4.0	4.5	4.5	2.07
Means	9.9 ± 1.9^b^	32.1 ± 3.6^a^	9.7 ± 1.7^b^	10.4 ± 2.1^b^	8.2 ± 1.6^b^	2.62 ± 0.16

1 Methods were: Alive, fixed = Alive broiler, with the femorotibiotarsal joint fixated; dead, non-fixed = Dead broiler, without the femorotibiotarsal joint fixated; Dead, fixed = Dead broiler, with the femorotibiotarsal joint fixated; Legs with muscles = Dissected legs, skin removed, but intact muscles and joints, with the femorotibiotarsal joint fixated; Legs without muscles = Dissected legs, skin and musculature removed, but intact joints, with the femorotibiotarsal joint fixated.

a,b Means within a line lacking a common superscript differ (*P* < 0.001).

## DISCUSSION

Aim of the current study was to investigate effects of different methods to measure VV angulation between the tibiotarsus and the tarsometatarsus of broiler chickens. Results clearly showed that in intact dead chickens without fixation at the femorotibiotarsal joint, the legs showed a large lateral deviation, resulting in an increased angle between the tibiotarsus and tarsometatarsus. At the moment that the leg was fixated at the femorotibiotarsal joint, the angle between the tibiotarsus and tarsometatarsus became much smaller and was strongly comparable with the angle in dissected legs with or without muscles (method 4 and 5). That a lack of fixation at the “knee” joint results in high levels of VV angulation is indeed demonstrated by Güz et al. (2019), showing that up to 70% of the chickens showed deviation from straight. Unfortunately, in studies using the method of Leterrier and Nys (1992) and Almeida Paz et al. (2010), it is not indicated whether or not fixation of the leg before VV angulation determination (visual or measuring) was used, meaning that comparisons can only be made within a study and hardly between studies.

It can be assumed that angulation of the dissected leg without muscles best represents the presence of VV angulation or not. In that case, only one out of six chickens (chicken 5) used in this study can be considered as having a mild VVD (above 10^o^ deviation), based on the classification of Leterrier and Nys (1992). However, it can be questioned whether or not their classification is valid for VVD in case legs are fixated during measuring the angle between the tibiotarsus and tarsometatarsus as done in the current study. This needs to be investigated with a higher number of chickens.

In the current study, no difference in VV angulation was found between the left and right leg, which is in accordance with Cruickshank and Sim (1986), Shim et al. (2012), Almeida Paz et al. (2013) and Guo et al. (2019). However, in another treatment group, Almeida Paz et al. (2013) showed higher VVD incidence in left legs than in right legs and Duff and Thorp (1985) and Riddell and Springer (1985) found higher incidence in right legs than in the left legs. These ambiguous results again suggest that objective measuring of VV angulation, using a fixed protocol is important to compare studies.

It can be concluded that the method used to determine VV angulation can affect the results obtained. Fixation of broiler legs at the femorotibiotarsal joint before determining the tibiotarsus – tarsometatarsal angulation seems to be important for correct measurement of the angle. It is suggested that fixated legs in combination with the measurement of the tibiotarsus – tarsometatarsal angulation is the most appropriate way to assess VVD in broiler chickens.

## DISCLOSURES

I hereby declare, on behalf of all authors, that we do not have any conflict of interest in relation to the study described in the manuscript “Comparing methods to assess Valgus-Varus deformity in broiler chickens.” The study described in this manuscript is executed according to the code of conduct of Wageningen University.
